# An ensemble micro neural network approach for elucidating interactions between zinc finger proteins and their target DNA

**DOI:** 10.1186/s12864-016-3323-9

**Published:** 2016-12-22

**Authors:** Shayoni Dutta, Spandan Madan, Harsh Parikh, Durai Sundar

**Affiliations:** 1 Department of Biochemical Engineering and Biotechnology, DBT-AIST International Laboratory for Advanced Biomedicine (DAILAB), Indian Institute of Technology Delhi, New Delhi, 110016 India; 2 Department of Computer Science and Engineering, Indian Institute of Technology Delhi, New Delhi, 110016 India

**Keywords:** Zinc finger proteins, Neural network, Statistical sampling, Targeted genome editing, Domain adaptation

## Abstract

**Background:**

The ability to engineer zinc finger proteins binding to a DNA sequence of choice is essential for targeted genome editing to be possible. Experimental techniques and molecular docking have been successful in predicting protein-DNA interactions, however, they are highly time and resource intensive. Here, we present a novel algorithm designed for high throughput prediction of optimal zinc finger protein for 9 bp DNA sequences of choice. In accordance with the principles of information theory, a subset identified by using K-means clustering was used as a representative for the space of all possible 9 bp DNA sequences. The modeling and simulation results assuming synergistic mode of binding obtained from this subset were used to train an ensemble micro neural network. Synergistic mode of binding is the closest to the DNA-protein binding seen in nature, and gives much higher quality predictions, while the time and resources increase exponentially in the trade off. Our algorithm is inspired from an ensemble machine learning approach, and incorporates the predictions made by 100 parallel neural networks, each with a different hidden layer architecture designed to pick up different features from the training dataset to predict optimal zinc finger proteins for any 9 bp target DNA.

**Results:**

The model gave an accuracy of an average 83% sequence identity for the testing dataset. The BLAST e-value are well within the statistical confidence interval of E-05 for 100% of the testing samples. The geometric mean and median value for the BLAST e-values were found to be 1.70E-12 and 7.00E-12 respectively. For final validation of approach, we compared our predictions against optimal ZFPs reported in literature for a set of experimentally studied DNA sequences. The accuracy, as measured by the average string identity between our predictions and the optimal zinc finger protein reported in literature for a 9 bp DNA target was found to be as high as 81% for DNA targets with a consensus sequence GCNGNNGCN reported in literature. Moreover, the average string identity of our predictions for a catalogue of over 100 9 bp DNA for which the optimal zinc finger protein has been reported in literature was found to be 71%.

**Conclusions:**

Validation with experimental data shows that our tool is capable of domain adaptation and thus scales well to datasets other than the training set with high accuracy. As synergistic binding comes the closest to the ideal mode of binding, our algorithm predicts biologically relevant results in sync with the experimental data present in the literature. While there have been disjointed attempts to approach this problem synergistically reported in literature, there is no work covering the whole sample space. Our algorithm allows designing zinc finger proteins for DNA targets of the user’s choice, opening up new frontiers in the field of targeted genome editing. This algorithm is also available as an easy to use web server, ZifNN, at http://web.iitd.ac.in/~sundar/ZifNN/.

**Electronic supplementary material:**

The online version of this article (doi:10.1186/s12864-016-3323-9) contains supplementary material, which is available to authorized users.

## Background

Zinc finger proteins are the most widely occurring transcription factors and have found applications in genome engineering [1]. The modular nature of zinc finger proteins has enabled custom design of these proteins for unique targets in any genome. However, the exact nature of zinc finger protein binding to its target DNA is not completely understood. Design of custom ZFPs for newer targets requires a better elucidation of the mode of interaction from a physico-chemical perspective.

Ab-initio prediction of a protein with optimal binding to any target DNA would be the paramount solution for therapeutic applications of genome engineering. Experimentally mapping protein-DNA interactions has seen considerable success [2], though the imperfections and cumbersome nature of high throughput experiments have limited absolute information about regulatory network for any organism, hence questioning the feasibility of these experiments. Computational tools affirming accurate and quick prediction of protein-DNA interaction can be the savior to fill this gap. The best prototype to propel development of such tools in the best interest of genome engineering is Cys_2_-His_2_ variants of zinc fingers. These transcription factors are well characterized and represent the largest class of DNA-binding proteins in metazoans.

Each finger of a ZFP, the most widely occurring transcription regulating factors, binds to a 3 bp DNA sub-sites i.e. the promoter region of the gene via the cardinal residues -1, +2, +3, +6 on its alpha helix. The specialty of the binding domains of this class of proteins is that they can be linked nearly in a tandem fashion to recognize nucleic acid sequences of varying lengths [3]. Zinc finger proteins which bind to four base pair DNA sub-sites via the “Recognition Code” on the alpha helix of each zinc finger, can be exploited to predict optimally binding ZFPs to any target DNA. Devising a method that analyses the physico-chemical properties of ZFP-DNA complexes and selects the most optimum zinc finger protein candidate for our target DNA by exploiting the relative strengths based on these interactions stands as the ultimate concern.

Zif-268 is a very useful model for studying zinc finger protein structure and function. Fusion of the recognition domain of tandemly linked zinc fingers to functional domains like nucleases, repressors [3] etc. bind to a very specific short nucleotide sequence around the major groove [4] whose statistical probability of occurring in the genome elsewhere by chance is low, hereby revolutionizing genetic engineering. This has many current applications in research and medicine such as repression of HIV expression, activation of expression of VEGF-A in a human cell line and the disruption of the effective cycle of infection of herpes simplex virus to name a few [3].

**Fig. 1:**
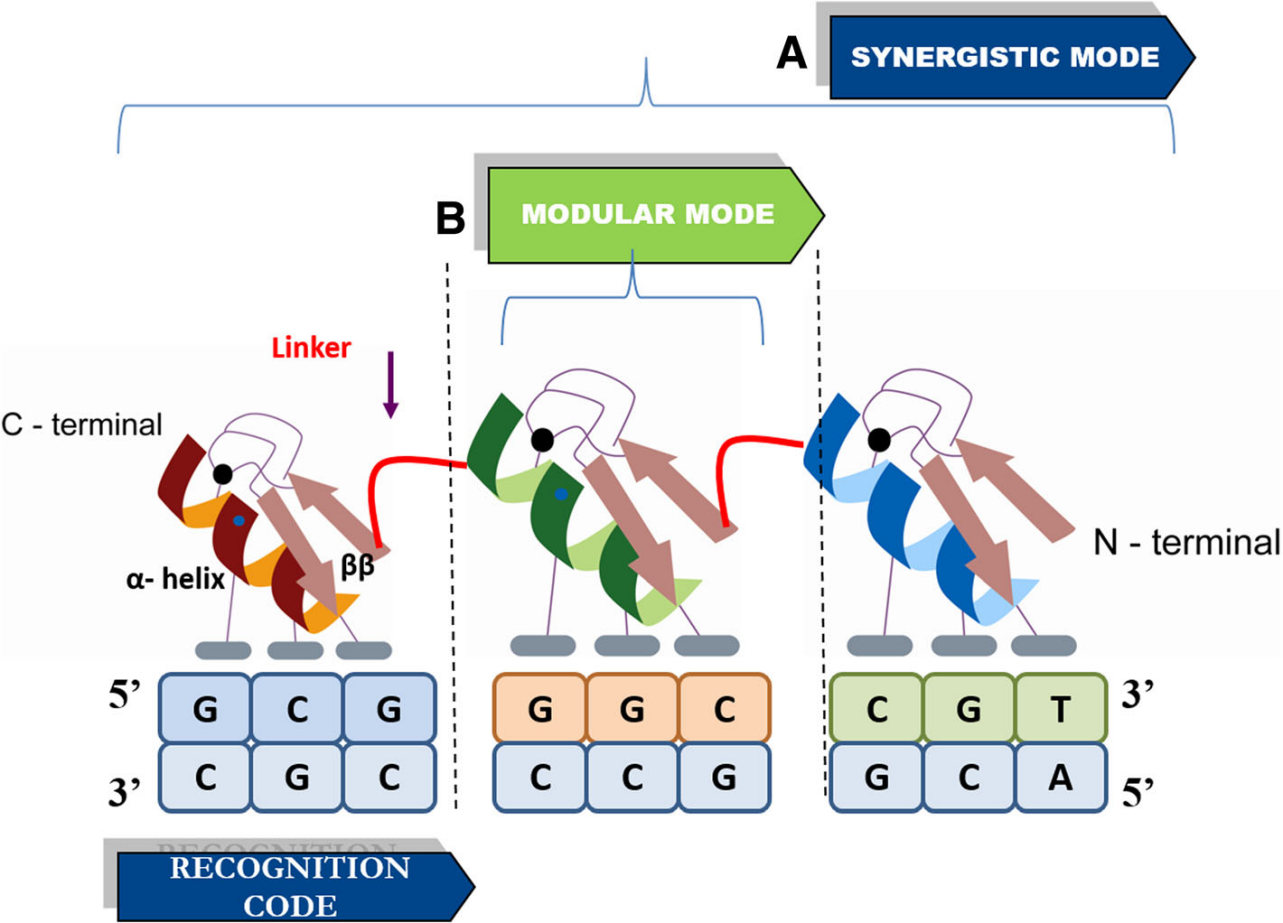
A schematic representation of DNA-zinc finger protein interaction depicting the two possible modes of binding. **a**) The binding affinity of each finger is affected by the adjacent fingers due to co-operativity - Synergistic mode of binding and **b**) Binding affinity of each finger with its respective 3 bp DNA sub-site is independent of each other - modular mode of binding

The binding of ZFP to its target DNA is assumed to have two hypothesized modes of binding: modular and synergistic. Modular mode of binding assumes that binding affinity of each finger of the protein is not affected by the other fingers (Fig. 1). The final energy for interaction between the target DNA and number of respective finger is additive energy of each finger. The advantage lies in individual investigation of each finger for its positional dependence and amino acid propensity ignoring the effect on affinity due to adjoining fingers. The disadvantage lies in dismissing this cooperative effect. Tools based on modular mode of binding: OPEN [5], *ZiFiT* [6], ZiF-Predict [7], ZifBASE [8]. These tools in addition to ignoring the cooperative effect of the zinc finger proteins, are unable to explore the whole sample space and predicts for a skewed sample space, which is GC rich. Hence, the need for a tool which does both and is able to predict with good accuracy when scaled for experimental datasets propels this research study.

**Fig. 2:**
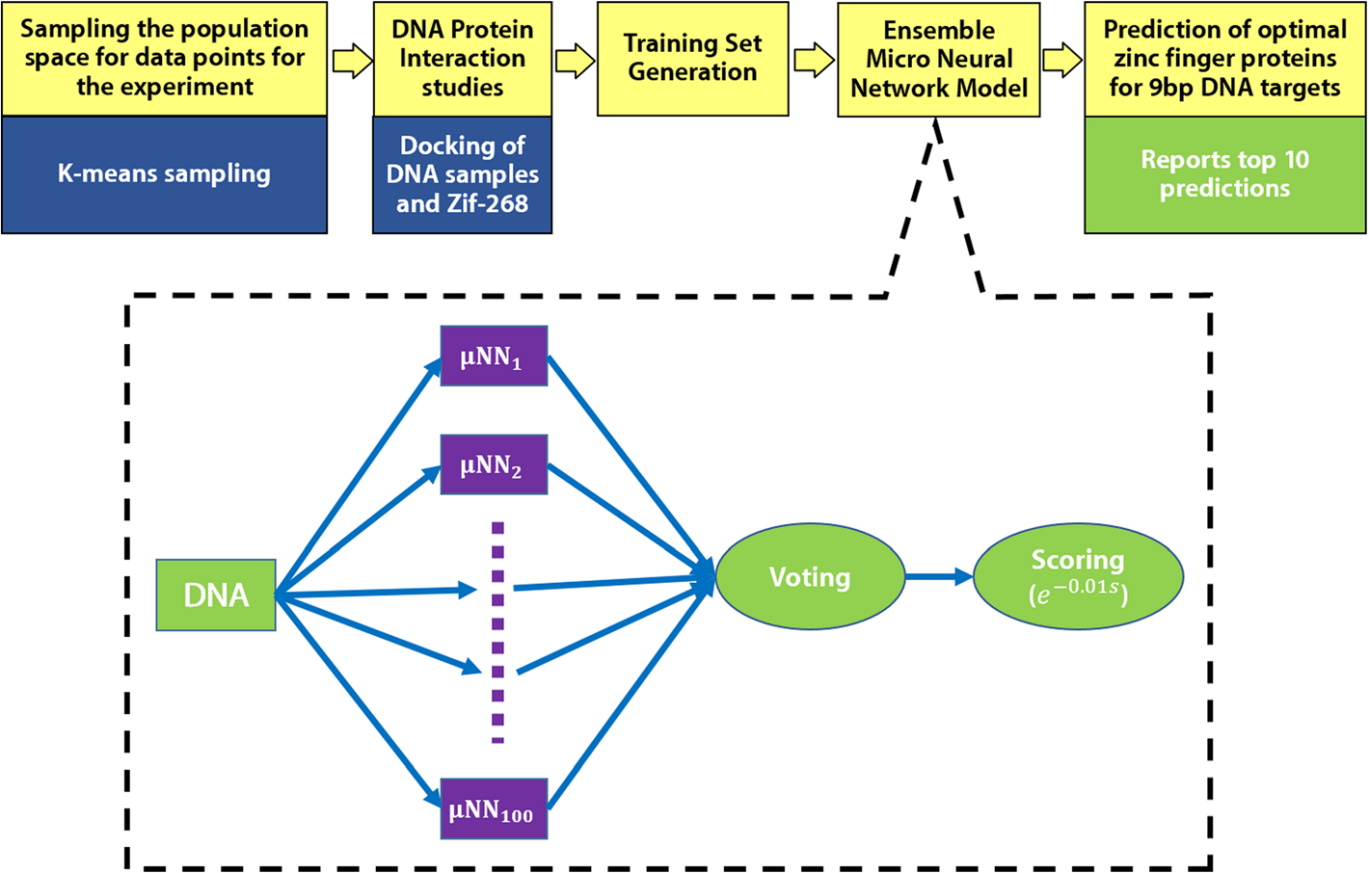
The pipeline for our algorithm to predict optimal ZFPs for any 9 bp target DNA. K-means sampling was used to identify sample points that represent the whole sample space well. These DNA samples are docked with mutants of the Zif-268 protein to generate the training samples for our ensemble micro neural network model. Finally, the model is used for making predictions for user queried 9 bp DNA targets

In synergistic mode of binding, the dependency of the fingers on each other is taken into account. Cross-strand interaction as well as the concept of co-operativity holds true (Fig. 1). The synergistic approach to ascertain the functioning of zinc fingers while interacting with the respective target DNA via their recognition code appears to be highly resourceful and reliable in terms of quantifying the physico-chemical interaction. This mode gives respite to the quandary whether the ideal mode of ZFP-DNA binding is modular or synergistic. The synergistic mode of binding is in a much closer to the natural ZFP-DNA binding. However, unfortunately in this case the individual fingers and their respective energies cannot be determined and evaluating all possibilities of an ideal three finger ZFP with its target 9 bp DNA is an impossibility in terms of both computational resources and time constraints. The problem at hand necessitates the need to develop an efficient predictive algorithm for predicting best binding proteins based on data obtained from docking and simulation strategies, which has proved to be credible upon validation with experimental datasets mined from literature. For this purpose, we relied upon a micro neural network (μNN) model in conjunction with the modeling and simulation data (Fig. 2). A μNN is defined as a micro neural network model, with the number of nodes in hidden layer typically of an order less than the dimension of output vector. The μNNs used for prediction have between 28 and 52 nodes.

The fields of biology and machine learning have been closely related for a long time now. The use of machine learning in biology has been reported in literature for solving problems pertaining to pattern recognition, classification, and prediction based on models derived from existing data [9]. The μNN, widely considered as a cornerstone in the field of machine learning had emerged from something known as the perceptron, which was an attempt to model the behavior of neurons in humans [10]. Towards the latter half of 2000, machine learning was actively being used in binding site predictions, primarily using sequence based features [11–15]. As more DNA-binding protein structures were identified through experimental work, the data available for prediction algorithms became richer in terms of possible features, opening up the gambit for a number of machine learning algorithms like ANNs [16, 17], Support Vector Machines (SVMs) [18, 19], Random Forest (RF) algorithm [14, 20] and Bayesian networks [12], and decision tree algorithms [15].

Mathematically, a neural network is a series of transformation matrices with a nonlinear operation after each transformation operation [21, 22]. Thus, the conceptualization of a neural network allows us to approximate the required transformation matrix by training the neural network with the true data [23, 24]. NNs have been shown to be successful in literature with even relatively smaller datasets [25–27]. Moreover, one distinguishing feature of NNs as compared to other machine learning techniques is the ability to extract features from the training set, a fundamental step in any machine-learning problem. There have been numerous studies in literature, which explore NNs as feature extractors for complex datasets [28–31]. Keeping these in mind, neural network was chosen as the preferred method for training the prediction model for our tool.

For high dimensional data; characteristic of our dataset, often a single ANN is not able to pick up all the relevant features, and thus, an ensemble μNN has been used to train the non-linear transformations relating the DNA sequence and its optimal binding ZFP in our tool. Ensemble μNN relies on the principle that multiple μNNs trained with the same dataset and different hidden structure differently approximate the needed nonlinear space transformation. Thus, the predictions made may vary from one μNN model to another, and the final result can be obtained by taking the consensus of these predictions [32, 33].

In our previous studies, we were able to draw correlation between binding affinity determined by docking scores and respective dissociation constant (K_D_) values from experimental data for the same sample. Complexes with lower K_D_ values mined from literature show stronger binding, which falls in sync with the finding that more negative docking scores showed higher binding affinity. Simulation studies for the same sample set affirm stability for complexes with higher binding affinity and more negative docking score [34]. Hence, we use this method to generate the most optimal ZFPs for the entire 50 sample DNA PDBs we have generated.

## Methods

### Protein and DNA sequences

The zinc finger skeleton used to start our pipeline was Zif-268 (1AAY). The cardinal residue positions (-1, 3 and 6) on the α-helix of Zif-268 interact with its corresponding 3 base pair DNA subsites which is the “recognition code”. We chose to work with Zif-268 as our starting skeleton because we have replete literature as well as the x-ray crystallography structure available for it [1]. Hence, it stands as the ideal prototype to propel our studies.

The DNA sequences that were used as our representative set of the whole sample space were generated using K-means clustering. The need for doing so arises from the fact that data reported in literature is highly skewed and GC rich. The training and the testing sample set DNA sequences have been documented (Table 1). These sequences were generated using CHIMERA in the PDB format [35].

**Table 1:** DNA Sequences used for training and testing of micro neural network Model

Training Sample Set												Testing Sample Set		
(Orientation 5′ → 3′)														
CGA	AAT	CGC	GCT	TAT	ACT	GCA	GCC	TTT	TTT	GCT	TCA	CAT	TTA	GTG
CAT	GTA	TGA	AGG	GCA	GCG	TAG	TCC	ATT	TTA	TTA	TGG	GGA	GGA	GGA
GTG	GCG	GGC	CCA	TAT	GCG	CTT	ACT	CTG	GGA	GCG	ATC	ACT	CAG	CTC
TAA	GCT	CAA	GTG	TAT	ATA	GCC	CAC	GAA	ACG	CAA	CAG	GGG	GGG	GGG
TGG	TGG	GGA	ACT	ACG	CTA	GAC	CCA	TAC	CGC	TTA	TTA	TGG	TGT	CCG
TCG	GCG	TGA	TAA	TGT	GGT	AGC	TAT	TTC	TCC	TCG	TGT	GTT	GTT	GTT
CAA	TCA	GAT	CCA	GAG	TCC	CGG	AGA	AGG	GTT	TCT	CTC	GCC	GCC	GCC
TGC	AAT	TGA	GTG	ATA	ATC	GCT	AGT	TAG	ACG	ATT	AGG	GCA	GCA	GCA
ACC	GAG	CTA	TTA	AGA	GAG	CGC	AGC	TAG	ATA	TTC	GAG	GAG	GAG	GAG
TGC	AGC	TAT	GAA	CGA	AGA	CCC	CAA	CTG	TTC	GGG	CAA	GGC	GGC	GGC

### DNA sequence dataset creation

Efficient sampling is a necessity for good prediction accuracy and scaling of a prediction model across all possible prediction cases [36, 37]. Sampling is a method to choose the subset of total population such that the sampled subset represents the population appropriately, encompassing the information pertaining to the diversity in the original population [38]. A common conjecture is that given a large enough sampled subset and an appropriate sampling methodology, information learned through a sampled sub-population can be close to that learnt from the whole population [39].

An optimal sample size was chosen taking into account the statistical margin of error, the confidence interval and the complexity of data point generation [40]. These points were selected from a population of size 4^9^ based on K-means clustering, where K = 50. K means clustering sampling reports the representative data point for each of the K clusters [41]. Assuming that there are pseudo-clusters of data points within the population space, we found a representative data point for each pseudo-cluster, thus obtaining a sub-population which is well representative of the whole population.

#### DNA-protein interaction studies

The HADDOCK software algorithm based on the data-driven approach, utilizes distance constraints extracted from experimental data (gathered from various possible sources, such as NMR, conservation data, etc.), to reconstruct and refine the protein-DNA complex. The docking is the most computationally heavy and time-consuming step, and thus had to be optimized. We assumed that the template (Zif-268) and the mutated protein differ at only certain key residues (at most 3 amino acids at the -1, +3 and +6 for the particular finger) and hence are not structurally too different which are used in indicating the active residues in HADDOCK. Therefore, in order to get a template complex structure with each DNA sequence, they were docked with Zif-268. The numbers of structures for rigid body docking (it0) were from 1000 to 750 and the number of structures for refinement (it1) were from 200 to 100 (rate determining step). There was no need to randomize the starting orientation of the protein before docking; hence, the parameter was set to False. This was justified as the structure of Zif-268 was extracted from its already complexed state with its consensus DNA and hence can be assumed to be close to the confirmation it would attain when docked with the new DNA. Solvated rigid body docking was not performed. The analysis we are conducting is without any solvent. The possible effect of the presence of a solvent like water, which might interfere with the intermolecular hydrogen bonding between DNA and protein, was discarded as it has been shown in literature that the effect of polar solvents on hydrogen bonding in DNA-protein complexes is minimal. The protein used to dock with each of the 50-DNA ensembles was Zif-268 (1AAY). Out of the numerous structures generated for each DNA-protein (Zif-268) pair, the structure with the greatest HADDOCK score was deemed the most suitable for that pair and further used in the next step.

#### Mutation of key residues in Zif-268

Excluding the residues that do not frequently function in DNA recognition helps reduce the library size and the “noise” associated with nonspecific binding members of the library. Therefore, the randomizations need not encode all 20 amino acids but rather represent only those residues that are most frequently found to occur in sequence-specific DNA binding from the respective α-helical positions (Additional file 1). With the help of data from [42], a list of most commonly occurring amino acids found at the key α-helical positions was prepared, listing the required mutations at key positions (Additional file 1). Mutating residues at positions -1, +3, +6 (keeping +2 fixed to eliminate cross strand interactions) using the listed amino acids in Additional file 1, the 7*8*8 possible recognition helices were considered and complexed with each DNA to finally rank the best helices for each codon.

In case the NMR or crystallographic structure of the protein is unavailable, homology modeling can be used to develop a reliable 3-D model for the protein if atleast one protein structure is available with some similarity to it. Therefore, homology modeling predicts the 3-D structure of a protein sequence of interest, the target relying on its alignment to one or more proteins with available experimentally determined 3-D structure called the template. Fold assignment, target-template alignment, model building, and model evaluation form the core of homology model prediction [43]. MODELLER, an open source tool used for comparative modeling aligns our target of interest to templates to automatically calculate a 3-D model for our target containing all non-hydrogen atoms [44]. Script was written and run which takes a particular template complex and depending on the finger under consideration (determined by the DNA sequence), performs mutations (Fig. 1.) to generate complexes with all possible recognition helices using MODELLER [45].

#### Determining hydrogen bonding parameters

To detect even single residue differences in the mutated recognition helices all the hydrogen bonding parameters like acceptor-donor distance and angles would need to be extracted from the PDBs. For this purpose, the LIGPLOT/HBPLUS software was used [46].

#### Calculation of free energy of hydrogen bonding

It has been found that amino acid–base hydrogen bonds are the most frequent interactions in protein–DNA complexes (50%), followed by van der Waals, hydrophobic, and electrostatic interactions [47].

A desirable and accurate rendition of the AMBER99 force field with its hydrogen bond energy component described below was used to calculate the free energy of hydrogen bonding. Once the target pairs were identified, the atom types (primarily N or O) of the donor and acceptor atoms were identified, the constants εij and dij’ values’ applied and the energy calculated. For a particular codon: helix file, the total hydrogen bond energy accounted for was the sum of individual energies of all specific pairs identified. The energy values for all helices for a particular codon (and finger) were saved as a database. The equation used to determine hydrogen bond energy:

$$ \varDelta \mathrm{G}\left(\mathrm{h}\mathrm{b}\right)=\in \mathrm{i}\mathrm{j}\ \left[3{\left(\frac{\mathrm{dij}\prime }{\mathrm{dij}}\right)}^8-4{\left(\frac{\mathrm{dij}\prime }{\mathrm{dij}}\right)}^6\right]{ \cos}^4\uptheta $$

Where εij is the optimum hydrogen-bond energy for the particular hydrogen-bonded atoms i and j, considering that d*ij is the optimum hydrogen-bond length. εij and d*ij vary according to the chemical type of the hydrogen-bonded atoms i and j. The above hydrogen bond energy function was used to quantify the DNA-protein interaction at the interface.

Assumptions:

- εij = 2.0 kcal · mol-1 and dij’ = 3.2 Å for N-N hydrogen bonds
- εij = 2.8 kcal · mol-1 and dij’ = 3.0 Å for N-O hydrogen bonds
- εij = 4.0 kcal · mol-1 and dij’ = 2.8 Å for O-O hydrogen bonds [48].

Each step was automated and a batch run was done using scripts.

### Details of the ensemble micro neural network developed

The 9 bp DNA sequence was encoded and represented as a vector of length 36, with a group of four dimensions representing a position in the DNA sequence – A as (1,0,0,0), T as (0,1,0,0), G as (0,0,1,0) and C as (0,0,0,1). A similar encoding was done to represent the Zinc Finger Protein of length 21 as a vector of length 420, each position of the protein represented by a group of 20 dimensions. The Neural Network models used had a sigmoidal thresholding after each matrix operation to approximate nonlinearity. Sigmoidal thresholding allows the output to be between 0 and 1 and thus conforms with the input–output representation. In the training phase, the objective is to minimize ||L||2 error on the output layer, by performing stochastic gradient descent. ||L||2 is a standard mathematical norm to measure an entity that corresponds to euclidean distance in real space. Minimizing ||L||2 between predicted and the actual output vector during training phase aims to minimize the euclidean prediction error in the transformed space. An ensemble machine learning approach utilizing100 Neural Networks in parallel was used, so as to minimize the modeling uncertainty. All the 100 Neural Networks were generated with single hidden layer and number of nodes in hidden layer of each neural network were randomly generated between 28 and 52. The neural network models are trained with 150 iteration of training dataset, shuffled after each epoch.

The model described above predicts the optimal protein. An ensemble of the results obtained by running each of the 100 neural network models on the user queried DNA sequence is reported as the best binding Zinc Finger Protein. For each position of the protein sequence, the amino acid which is predicted by the maximum number of ANN models is reported as the most appropriate amino acid at that position.

$$ \begin{array}{c}\hfill Sigmoid(x)=\frac{1}{1+{e}^{-x}}\hfill \\ {}\hfill LayerOperation(X)= Sigmoid(W.X)\hfill \end{array} $$

Where x is the input and W is the weight matrix for the transformation function.

### Scoring function

The quantification of the accuracy of a prediction made by our algorithm is done by a scoring function, which ensures appropriate resolution amongst the predictions. The score value is calculated for each prediction as the negative exponential of the sum of total number of votes the protein sequence gets for each position. A more negative exponent implies better prediction confidence on the result, thus the score value will be smaller for better predictions. As the voting is done for each position, using an exponential will convert an addition of the votes to multiplication of exponential terms, thus, if the confidence at a particular position is low, it will reflect strongly in the score.

$$ \begin{array}{c}\hfill Accuracy\  Score = {e}^{-0.01s}\hfill \\ {}\hfill \begin{array}{cc}\hfill \mathrm{Where},\hfill & \hfill s = {\displaystyle \sum_{i=1}^{21}}\mathrm{N}\mathrm{o}.\ \mathrm{o}\mathrm{f}\ \upmu \mathrm{N}\mathrm{N}\ \mathrm{which}\ \mathrm{voted}\ \mathrm{f}\mathrm{o}\mathrm{r}\ \mathrm{the}\ {i}^{th}\ \mathrm{position}\ \mathrm{o}\mathrm{f}\ \mathrm{predicted}\ \mathrm{protein}\hfill \end{array}\hfill \end{array} $$

In order to optimize the number of predictions that our algorithm reports, the relationship between the number of predictions reported, and the best prediction accuracy for the testing dataset was closely studied. It was seen that the graph between the two approached a plateau as the number of predictions reported approached 10, and that there was no significant improvement in the best prediction accuracy after that. Thus, ZifNN reports the top 10 predictions for a user queried DNA sequence.

## Results and discussion

### Validating the binding affinity for our training sample set

The HADDOCK scores based on our previous study adhere to the inference that more negative the docking score, higher the binding affinity [34]. The study also confirmed that score around or more than -140 show very high binding affinity. Hence, the average docking score for the sample ensemble is -151.287, which indicates good and reliable docking scores. Thus, the part of our pipeline that includes docking was run successfully with good precision.

After docking, the pipeline generates hydrogen bond energies for each sample and its optimal binding ZFPs. The hydrogen bond energy for the 50-data ensemble for their top binding ZFPs has an average of -6.814. To validate the effect of the energy change due to hydrogen bonding, a small sample set was run through the same algorithm and the results compared to experimental data of helix QNK [49]. Lower the K_D_ value higher the binding affinity, which translates to more negative or lower value of free energy change due to hydrogen bonding showing higher affinity as well. We validated that the energy change for finger 2 of our predictions was in coherence with the experimental data for the helix type QNK [49].

The success of the above two steps of our algorithm lies in their validation based on data mined from literature assuring their reliability. This algorithm cannot be run for all possibilities i.e. (4)^9^ [all possibilities of a 9 bp DNA] * (448) [mutations for all three fingers of Zif-268], hence we opt for machine learning. Accuracy in validation at these crucial stages paves way to adopt an approach employing a prediction model based on machine learning with high confidence.

### Accuracy of the ensemble micro neural network prediction model

One of the guiding principles in the field of bioinformatics is the notion that sequence similarity, albeit loosely, is related to functional similarity. Sequence identity is widely used as measures for sequence comparison [50, 51]. Thus, Sequence identity was used as one of the metrics to measure accuracy of our predictions, which was measured a position-wise comparison of the predicted sequence with the optimal sequence and reporting the percent of positions which matched with the optimal protein. Mathematically, this measure is a variant of Hamming distance, which is a widely used string metric [52]. However, it has often been contended that homology, and thus function departs very quickly with departing sequence identity. In order to account for this, we have also reported the average BLAST e-value for the testing sample set (Table 2) [53].

**Table 2:** Accuracy of micro neural network model for both the training and testing datasets (Sequence Identity and BLAST e-value scores)

	Training Data	Testing Data
Median BLAST e-value score	2.00E-21	7.00E-12
Geometric Mean of BLAST e-value scores	3.00E-21	1.70E-12
Average Sequence Identity	100%	83%

The 50 data point sample set was divided into two subsets of 40 and 10, former was used for training while latter was used for testing the model and its generalizability across other datasets. The training dataset was used to train the neural network ensemble model. To test the performance of model and to check over-fitting, the testing set was used on the trained model [54].

### Domain adaptation: validation with experimental data

Final validation of our algorithm was done by comparing its predictions against experimentally identified best binding ZFPs for DNA sequences which have been studied experimentally [55]. This approach, based on the idea of domain adaptation, was used to estimate its accuracy on data reported in literature. Domain adaptation is the ability to use the features learnt from data points belonging to a particular domain to predict results for data points belonging to a different, but closely related dataset [56]. For the purpose of our algorithm, the neural network was trained with a diverse, but representative set of the entire space of 9 bp target DNA sequences, while its validation is done on experimental data obtained from literature.

We have catalogued a list of over 100 9 bp DNA targets and their optimal zinc finger binding proteins and their respective K_D_ values, which have been reported in literature [57–66] (Additional file 2). The metric chosen for validation of our predictions with the catalogue of experimental data was string identity calculated as the Hamming distance between the experimentally identified alpha helices and the helices predicted by our tool. The average identity for our predictions as compared to the experimental data in the catalogue described above was found to be 71% (Additional file 3).

### Positional preference for DNA binding specificities: an observation

The accuracy of our algorithm, as measured by the average string identity, was found to be as high as 81% for DNA targets with a consensus sequence GCNGNNGCN reported in literature. However, for DNA targets with a consensus sequence GNGNA/TNGAN was found to be around 62%. The consensus sequences for the same were obtained using CLUSTALW2 [67].

### Comparison with other tools

A number of other tools have been reported in literature which attempt to predict optimal zinc finger binding protein for a target DNA sequence. However, most of these are based on algorithms assuming modular binding between the target DNA and its respective zinc finger protein. As synergistic binding takes into account the co-operativity of zinc finger binding affinities, it comes closest to mimicking the molecular interactions found in nature. Thus, the predictions made by our algorithm are much more biologically relevant. This was confirmed when we compared the predictions made by our tool to others found in literature including *ZiFiT* [68] and *Zinc Finger Tools* [69] (Table 3). Moreover, other tools based on synergistic binding reported in literature have not covered the whole sample space of 4^9^ DNA sequences. Thus, they are not able to predict optimal ZFPs for all possible user queried DNA target sequences.

**Table 3:** Comparison of ZifNN predictions with other tools reported in literature. ZiFNN, ZiFiT [6] and Zinc Finger Tools [4] were compared with experimental data mined from literature (K_D_ and helix prediction)* using Hamming distance as the metric

DNA Target 5′ → 3′	References from Literature	Experimentally Found ZFP			Best prediction made by ZiFNN	Identity for ZiFNN	ZiFit Prediction			Identity for ZiFiT	Zinc Finger Tools			Identity for Zinc Finger Tools
		F1	F2	F3			F1	F2	F3		F1	F2	F3	
GTGGAGGAA	[57]	QSGNLTRRSGHLTRRSGELTR			DSGHLTRDSGHLTRDSGHLTR	0.76	RNVNLVTRQDNLGRQASNLLR			0.33	RSDELVRRSDNLVRQSSNLVR			0.47
GCTGCTGCT	[58]	RSGELTRTSGELTRRSGELTR			TSGELTETSGELTETSGELTE	0.76	LRASLRRQRSDLTRMKNTLTR			0.38	TSGELVRTSGELVRTSGELVR			0.76
GAGGAGGAT	[59]	QSGNLTRRSGNLTRRSGNLTR			QSGHLTRQSGHLTRQSGHLTR	0.76	-			-	RSDNLVRRSDNLVRTSGNLVR			0.66
CTGGCGGCA	[60]	RSGALTERSGDLTRQSGDLTR			RSGDLTTRSGDLTTRSGDLTT	0.76	-			-	RNDALTERSDDLVRQSGDLRR			0.76
GGGGCGGGG	[61]	KSGHLTARSGELTRRSGHLTK			RSGHLTRRSGHLTRRSGHLTR	0.80	RKHRLDGRTDTLARRGNHLRR			0.33	RSDKLVRRSDDLVRRSDKLVR			0.42
GCTGGGGGC	[62]	RSGELTRTSGHLTRDSGHLTR			QSGHLTRQSGHLTRQSGHLTR	0.80	VSNSLARRREHLVRTNSKLTR			0.42	TSGELVRRSDKLVRDPGHLVR			0.61
GCGTGGGGA	[63]	RSGELTRRSGHLTRQSGHLTR			QSGTLTRRSGTLTRQSGTLTR	0.80	-			-	RSDDLVRRSDHLTTQRAHLER			0.61
GCGTGGGCA	[64]	RSGELTRRSGHLTRRSGELTR			RSGTLTRRSGTLTRRSGTLTT	0.80	-			-	RSDDLVRRSDDLVRQSGDLRR			0.57
GCGTGGGAA	[63]	RSGELTRRSGHLTRQSGNLTR			RSGTLTRRSGTLTRRSGTLTR	0.80	-			-	RSDDLVRRSDHLTTQSSNLVR			0.66
GCGGGCCGC	[65]	RSGELTRDSGALTRRSGELTR			RSGHLTRRSGHLTRRSGHLTR	0.80	-			-	RSDDLVRDPGHLVRHTGHLLE			0.47
GCAGCGGAC	[62]	RSGELTRRSGHLTRQSGSLTR			QSGHLTRQSGHLTRQSGHLTR	0.80	QKGTLGRRTDTLARDPSNLIR			0.38	QSGDLRRRSDDLVRDPGNLVR			0.52
GAGGAAGGG	[59]	RSGHLTRQSGNLTRRSGNLTR			QSGHLTRQSGHLTRQSGHLTR	0.80	RRDNLNRQQTNLTRKRERLDR			0.48	RSDNLVRQSSNLVRRSDKLVR			0.61
ACTACTGGA	[60]	TSGDLTRTSGDLTRQSGHLTR			TSGELTRTSGELTRTSGELTR	0.80	-			-	THLDLIRTHLDLIRQRAHLER			0.57
GCTGGGGGC	[62]	QSGDLTRRSGHLTRDSGHLTR			QSGHLTRQSGHLTRQSGHLTR	0.85	VSNSLARRREHLVRTNSKLTR			0.48	TSGELVRRSDKLVRDPGHLVR			0.61
GAAGAGGGT	[59]	QSGHLTRRSGNLTRQSGNLTR			QSGHLTRQSGHLTRQSGHLTR	0.85	QRNNLGRRQDNLGRTRQKLET			0.38	QSSNLVRRSDNLVRTSGHLVR			0.61
GAGGAAGGT	[66]	TSGHLTRTSGHLTRRSGELTR			TSGHLTRTSGHLTRTSGHLTR	0.90	RRDNLNRQQTNLTRTKQRLEV			0.28	RSDNLVRQSSNLVRTSGHLVR			0.47
					Average for ZifNN	0.81	Average For ZiFit			0.38	Average for Zinc Finger Tool			0.58

The average identity for predictions made by ZifNN was found to be 81% for DNA targets with consensus sequence GCNGNNGCN. *ZiFiT* was able to report the optimal ZFP for only 56% of the queried DNA targets [68]. The average identity of the predicted helices for *ZiFiT* was found to be 42%. Though, *Zinc Finger Tools* was able to report the optimal ZFP for all the queried DNA targets, the efficiency was found to be only 58% [69].

Moreover, for majority (82%) of the sample set used for comparing ZFP prediction tools, the K_D_ value was found to be <0.5, indicating high confidence in the annotation of their DNA binding specificities. This shows that ZifNN is capable of domain adaptation and makes biologically relevant predictions, which scales well to experimentally validated zinc fingers with higher confidence than other tools reported in literature.

## Conclusion

Zinc finger proteins have proven to be indispensable tools for targeted genome editing. While there are a number of approaches reported in literature to predict optimal ZFPs for target DNA sequences, they have had limited success in doing so with high accuracy. This can largely be attributed to two major factors – Firstly, most tools fail to capture the co-operativity of subsequent zinc finger binding affinities by assuming modular mode of binding. While there have been disjointed attempts to make predictions assuming synergistic mode of binding reported in literature, there is no tool which does so for the whole sample space of all possible 9 bp DNA targets. Secondly, the datasets reported in literature are highly GC rich, and are thus, a skewed representation of the whole sample space. Thus, tools based on learning features from experimentally reported data alone are not generalizable to the whole sample space.

We present here a novel algorithm combining an ensemble micro neural network in conjunction with domain adaptation to make predictions about DNA-Zinc Finger Protein binding specificities to overcome the above mentioned hurdles plaguing the tools currently existing in literature. Our algorithm assumes synergistic mode of binding, thus capturing the molecular interactions between the DNA sequence and the ZFP helices in greater detail. The exponential increase in the number of possible complexes is accounted for by using a small, but diverse sample set which well represents the whole space of possible DNA targets to train an ensemble micro neural network model, which is then used to make predictions about the rest of the dataset.

Moreover, our micro neural network is capable of domain adaptation, which allows it to make predictions about data points from a domain other than the one used for training the model. This enables us to make predictions with much higher accuracy for the DNA sequences that are not GC rich as well. This was confirmed by the comparative analysis of our tool against others reported in literature.

Using domain adaptation in conjunction with machine learning comes across as a powerful tool which can be exploited in biology, which is characterized by small, high dimensional datasets which are skewed and not well representative of the whole sample space. Our algorithm promises to opens new frontiers in the field of targeted genome editing, by enabling the scientific community to design zinc finger binding proteins for DNA targets of their choice. It’s implementation in the form of the ZifNN web-server is easy to use, and reports top 10 predictions for the user along with an accuracy score reflecting the biological significance of the prediction.

## Additional files

Additional file 1: List of most frequently occurring amino acids at the key positions like -1, 3 and 6 of the α-helix of the ZFP. (PNG 73 kb)
Additional file 2: Validation of ZifNN predictions by comparison with experimental helices. The Hamming distance between the catalogue of experimentally determined helices and the helices predicted by our tool are reported for different target DNA sequences. The average identity for these predictions is about 71%. (XLSX 79 kb)
Additional file 3: Evaluation within our top predictions for any given target DNA sequence. Analysis for the top 10 predictions for each experimental DNA target and their comparison based on e^-s^ score for each prediction. Further string identities have also been calculated to check the variation between the top 10 predictions for each DNA target. (XLSX 12 kb)

## Abbreviations

ZFP: Zinc finger proteins
μNN: Micro neural network
