# Supplementary figures and images for: An ensemble micro neural network approach for elucidating interactions between zinc finger proteins and their target DNA
Source: BMC Genomics. 2016 Dec 22;17(Suppl 13):1033. doi: 10.1186/s12864-016-3323-9 (PMC5260015; doi:10.1186/s12864-016-3323-9)

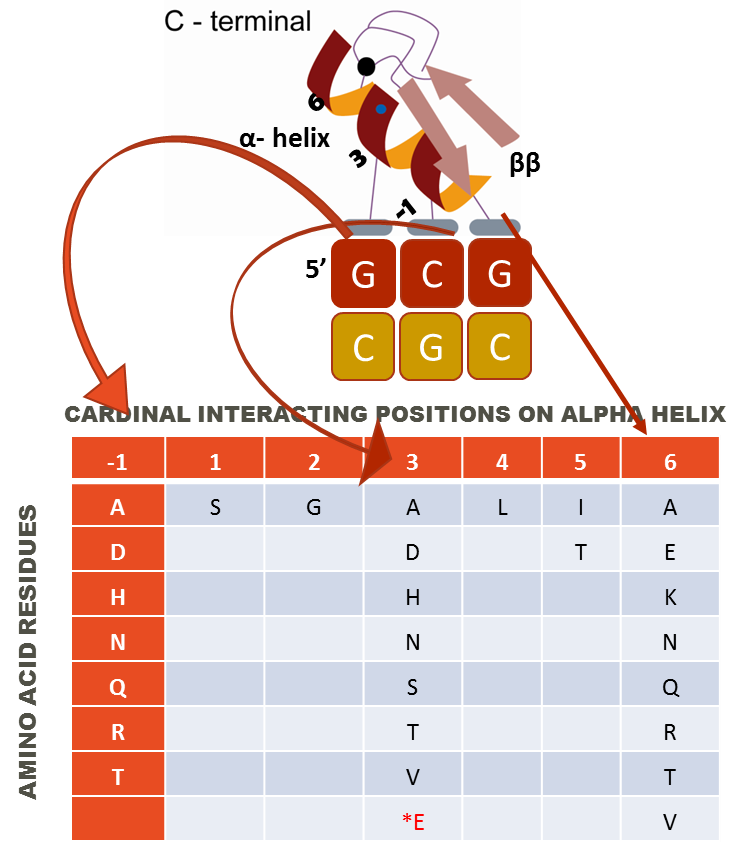

Supplement: Additional file 1: — List of most frequently occurring amino acids at the key positions like -1, 3 and 6 of the α-helix of the ZFP. (PNG 73 kb) [file 12864_2016_3323_MOESM1_ESM.png]
